# Network Intervention, a Method to Address Complex Therapeutic Strategies

**DOI:** 10.3389/fphar.2018.00754

**Published:** 2018-07-12

**Authors:** Chi Zhang, Wei Zhou, Dao-Gang Guan, Yong-Hua Wang, Ai-Ping Lu

**Affiliations:** ^1^Institute of Basic Research in Clinical Medicine, China Academy of Chinese Medical Sciences, Beijing, China; ^2^School of Chinese Medicine, Hong Kong Baptist University, Kowloon Tong, Hong Kong; ^3^The College of Life Sciences, Northwest University, Xi’an, China

**Keywords:** network intervention, therapeutic strategy, complex disease, network pharmacology, algorithm

## Abstract

**Objective:** Network-based approaches emerged as powerful tools for studying complex diseases. Our intention in this article was to raise awareness of the benefits of new therapeutic strategy in biological networks context and provide an introduction to this topic.

**Methods:** This article will discuss the rational for network intervention, and outline some of the important aspects of deciphering targets activities in the network and future embodiments of network intervention. We also present examples of network intervention based on the strategies these approaches use.

**Results:** Network intervention seeks for target combinations to perturb a specific subset of nodes in disease networks to inhibit the bypass mechanisms at systems level. Experimental results derived from our studies are discussed, with conclusions that lead to future research directions. A simple diagram is designed to give a way to find the minimum number of external input required for a network intervention based on the graph theory and get the analytical value of the least input.

**Conclusion:** Creating network intervention that addresses blindness and unthinking action in this way could, therefore, provide more benefit than multi-target therapy. We hope that this article will give readers an appreciation for a new therapeutic strategy that has been proposed for improving clinical benefit by adopting network-based approaches as well as insight into their properties.

## Introduction

Contemporary classification of disease systems has properties that limit its usability in some situations, especially therapeutic strategies. Thus a new approach network classification has been proposed to classify human disease status (Lu et al., 2008; Zhang et al., 2016). This classification method became more precise because of the network approach gained an increasing ground in modeling biological processes by now. Complex networks have progressed steadily to provide a specially promising framework for systems biology investigation. Further research is needed to shed more light on important practical applications related to properties of networks, in particular about therapeutic approach, therapists have unprecedented opportunities to increase their value and significance. For this purpose, it may be assumed that network intervention can be an effective approach. Network intervention is the application of network science toward modifying clinical outcomes in patients. Accordingly, this paper analyzes network intervention in biological processes in light of this strategy.

### The Limitation of Traditional Clinical Disease Classification

Traditional approaches of clinical disease classification have been based on pathological analysis and existing knowledge of diseases (Robinson, 2012). However, traditional diagnostic approaches are prone to errors (Wu and Montgomery, 2008; Loscalzo et al., 2016). Consequently, discover a potential new therapeutic strategy for difficult-to-treat diseases is beset with problems (Schreiber, 2000), given the lack of reliable pathogenic criteria. To help identify physiological failures, diseases can be defined as specific sets of phenotypes affecting one or multi-systems (Goh et al., 2007; Hidalgo et al., 2009). This explains why often current therapeutic options are single- or multi-target directed. However, the current state of knowledge about target directed strategy which based on matching disease classification is far from enough. Although most diseases are often treated separately, they are not independent of each other (Menche et al., 2015). There are no clear boundaries between many diseases (Rosenberg, 2006), as diseases can have multiple causes and can be related through several dimensions (National Center for Complementary and Alternative Medicine, 2004; Hidalgo et al., 2009; Barabási et al., 2011). Network approach may provide a systems level understanding of human status complexity (Zanzoni et al., 2009).

### Network-Based Classification to Diseases

During the past half-decade, network-based classification to diseases has undergone a revolution because of the emergence of new theoretical tools and techniques (Csermely et al., 2005). This further understanding makes us to view diseases as the network than as single gene. Meanwhile, evolving therapeutic strategies to improve outcomes of patients were discussed. Given the multi-components of networks, single component often fails to restore disease inducing systems failure (Tun et al., 2011). And sometimes the effects of drugs with high doses can spread throughout the network in which they act, causing some unwanted adverse reactions. From single- to multi-target, medicinal chemists still rely on some version of lock–key paradigm to the design of novel therapeutics. Now they recognizes that final effects of a given drug on a biological system may depend not only on the specific ligand-target recognition events but also on the influence of a specific intervention on human body systems.

### Potential New Therapeutic Strategy: Network Intervention

**FIGURE 1:**
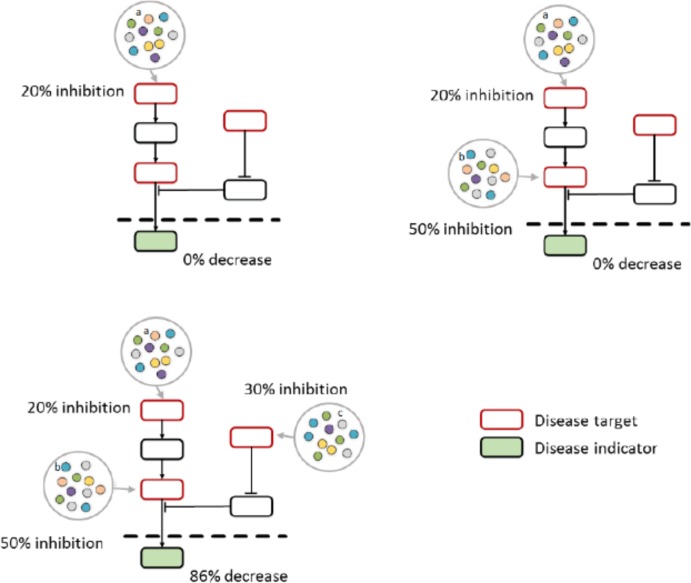
The network model of pharmacological actions.

In such a network context, therapy response can be considered from robustness of human status networks to deal with node attacks, due to inherent diversity and redundancy of compensatory signaling pathways that result in highly resilient network system with interconnected topology. Therefore, network intervention seeks for target combinations to perturb a specific subset of nodes in human status associated networks to inhibit the bypass mechanisms at systems level. Network intervention can help therapists to better understand why sometimes the effect of targeted therapies varies heterogeneously even if they directly bind to the molecules. Although direct binding, no clinical indicator is visually effective. Networks can be attacked after intervention in many ways most links in networks are weak, observed changes will be given to clinical indicator. In a network model of pharmacological actions, red elements of the network represent various targets (**Figure 1**), the drug candidate molecule binds to its target, which is a part of a network. The effect of activation of a target (regulators a), which is the usual outcome of the current single target drug design paradigm, is shown. The effect of regulator 1 is inhibited 20%. Again, another target (regulators b) can go into partial effect, inhibited 50% is shown. Unfortunately, this induced top-down effect on disease indicator shows ineffective. Finally, regulators a–c, act together resulting in the development of efficient network interventions and 86% decrease in disease indicators. In this case, a target-sets pattern intervention might be sufficient to achieve a significant modification.

### What Are the Difference Between Network Intervention and Multi-Target Intervention?

-1The biggest difference between network intervention and multi-target intervention is the way of building of effects with/without network science. Multi-target intervention focus on multi-target recognition ability and network intervention quests for the effect of target combinations to perturb a specific subset of nodes in disease network. From multi-target perspective, combination interventions are the standard of care for the treatment of diseases including cancer, T_2_DM, viral and bacterial infection, and asthma (Zimmermann et al., 2007). Plants also developed a combinative-strategy (Kubinyi, 2003). Multi-target effect is considered overcome the adverse reactions associated with high doses of single drugs by countering pathway compensation and thereby increasing effect while minimizing overlapping toxicity and allowing a lower dose of each compound. Actually, recognizing that positive target effects change *in vivo* is not a matter of choosing more targets, but choosing appropriately perturbing function. For example, Isorhamnetin, Nardostachin, and Jatamansin are the three active ingredients of Chinese herbal medicine CDF. Enoxaparin sodium, a low-molecular-weight heparin prepared from porcine intestinal heparin, is used for the prevention and treatment of venous thromboembolism (Buckley and Sorkin, 1992). Conventional scientific understanding of multi-targets is that there are more reliable targets were hit by the action of the three ingredients, but our research suggests the antiangiogenic effect of enoxaparin sodium (normal concentration) was significantly higher than that of three ingredients of CDF (**Supplementary Figure S1**). This example shows that network intervention strategies not myopic view only with targets combination but perturbing ability of drug for disease network. From single target to multi-target therapies, those paradigms have achieved considerable success in some diseases.

Now, we are in turning points in treatment of the disease they have failed to provide an effective approaches (Talevi, 2015). Network intervention may offer hope for an effective treatment. In this paper, we will discuss the rationale for network intervention, and present a strategy to develop modeling approaches to identify targets in a network context that could be leveraged for therapeutic benefit.

## Rationale

### The Characteristics of Network Intervention

Networks are characterized by a number of highly connected nodes while most nodes interact with a few neighbors (Alm and Arkin, 2003). These highly connected nodes have been proposed to play important roles in biological processes and shown to be related to the modular structure of networks (Liu et al., 2007; Sejoon et al., 2011). Therefore, it might be interesting to consider complex disease related key nodes (targets) in the context of the self-organized properties of interaction networks. Self-organized criticality is unstable state of networks in which tension develops as the network grows (Akker et al., 2011; Janina and Thilo, 2014). The tension is released by an avalanche type change in the network when the system becomes critical, because many of its elements behave identically as in transition phase (Dancík et al., 2010). The probability of this critical behavior often follows a power law and concentration threshold (Sanatkar et al., 2015). In some cases, network intervention effect can be obtained from different targets combination. Sometimes they are just like teams trying to reach the same destination via different paths. Often, single target node selection in dynamic networks is more difficult for choosing a deterministic node applies in different subjects. Consequently, many single-target drugs are not fully correct complex conditions such as cancer. By contrast module nodes in network with low concentration sometimes satisfy an important structural property. Once concentration threshold of module nodes is reached, the effects of target-sets pharmacological activities recoveries can lead to disease network reverts to its original state and avoid overreaction. Certainly, concentration threshold and different combinations are the parameters in considering the effect of network interventions.

### More Than One Best Intervention Can Be Determined? A Network Modeling Example

**FIGURE 2:**
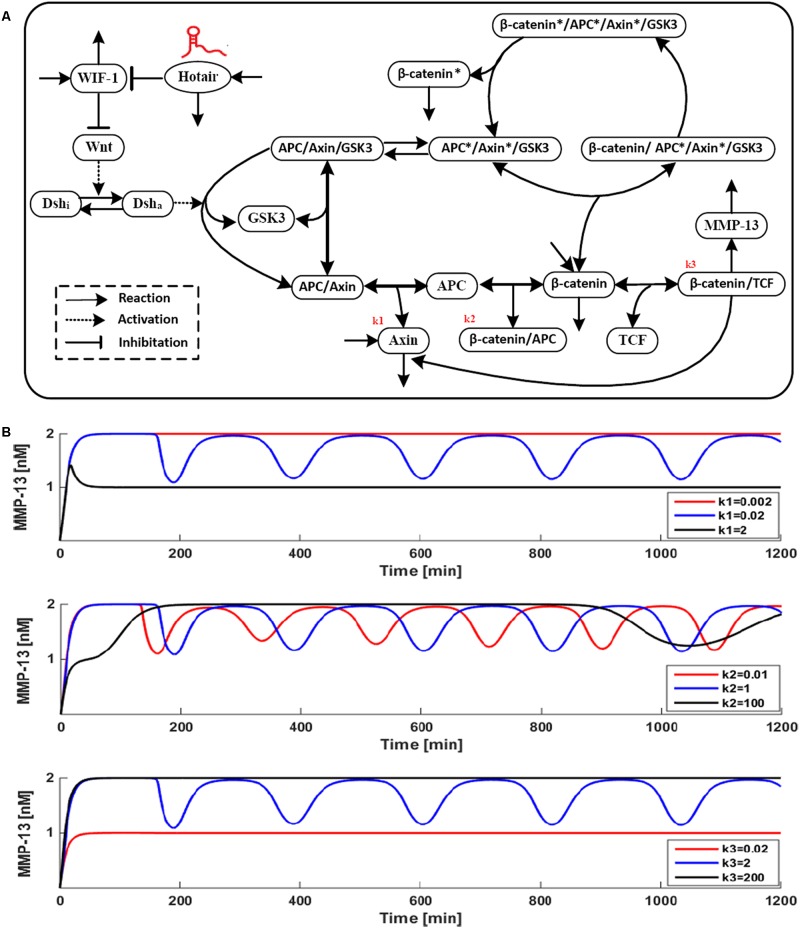
**(A)** Reaction scheme for a model of the Wnt pathway. **(B)** Effect of Axin, APC/β-catenin, and β-catenin/TCF on MMP-13 dynamics behavior.

To recognize that network modeling approach provides an opportunity to understand the complex relationships and direct quantitative analysis of dynamic network, and show directly the effects of combination intervention, we generated network model such that it with parameter variation. Wnt signaling (Clevers, 2006), one of the most critical signaling pathway in rheumatoid arthritis (RA) pathogenesis, is a potent pathway to regulate the expression of matrix metalloproteinase-13 (MMP-13) (Wernicke et al., 2006), while MMP-13 is known to be one of key factors responsible for degradation of collagen type II in articular cartilage. Wnt signaling is initiated by targeting the “destruction complex” consisting of the core scaffolding proteins Axin and APC that promotes the phosphorylation of β-catenin. And stabilized β-catenin translocate to the nucleus and functions as a co-factor of TCF transcription factors to trigger the transcription of Wnt target gene (Sen, 2005). A mathematical model for Wnt/β-catenin dynamic network regulating MMP-13 was developed by employing a chemical kinetic reactions approach, as illustrated in **Figure 2A**. **Figure 2B** shows a time series of the dynamic behavior of major components in Wnt/β-catenin pathway. The changes of single or multi-parameters related to Axin, APC/β-catenin and β-catenin/TCF induce the effects on the MMP-13 dynamic behavior of our model was a detailed analysis. The values of three most sensitive kinetic parameters k1–3 were set to increase or decrease by 1200×, with the other parameters fixed. The obtained results are shown in **Figure 2B**, in which the blue, red, and black curves represent the variation of k1–3, respectively. As seen in **Figure 2B** (1), the value of k1 was set to 0.0002, 0.02 (‘basal’ value) and 2 nM min^-1^. When k1 increases from 0.02 to 2 nM min^-1^ (black curve) or decreases from 0.02 to 0.0002 nM min^-1^(blue curve), the oscillations of MMP-13 disappear and subsequently reach steady state. Since k1 is the synthesis rate of Axin that is a suppressor of Wnt/β-catenin pathway, the concentration of MMP-13 decreases when k1 rises to 2 nM min^-1^. Compare with Axin (Kishida et al., 1998), a reliable and key target which should now be regarded as a tumor suppressor, the effects of two targets combination APC/β-catenin and β-catenin/TCF with low concentration are extremely similar to Axin. It is interesting to observe that different targets combinations seem to have obtained the same effect independently. This example also shows how the effect of multi-parameters causes changes in the dynamic behavior of network model.

### The Rationale for Network Interventions Can Be Especially Applicable to Oncology

The combination approaches aim to discover the unknown off targets for the existing drugs. Clinically efficacious cancer therapies often multi-target because the process of oncogenes is known to be multi-genic, and most cancers have some independent mutations (Tang and Aittokallio, 2014). These tumorigenic viruses use a multi-target approach to drive their own proliferation. Given that multiple nodes in the network must be modified to induce cancer, recently many researchers have proposed that multiple interventions will be required to counter this process. A much anticipated is try to find strongest one of all possible drug combinations. In this specific case, we aimed to find the most effective drug combinations. Chemotherapy drugs including GEM, VIN, DOX, ETO, CIS, and ASP, known to be clinically effective against cancer and selected by algorithm, they were mixed by the following methods: single drug (6 possible ways), two drugs mixed (15 possible ways), and three drugs mixed (20 possible ways). The drug combinations were incubated *in vitro* with the SKOV3 cells for inhibitory efficacy evaluation. The concentration of each drug in the system was equal to the critical concentration, which was obtained in our previous experiment. Finally, the inhibitory efficacies of two combinations (GEM+VIN+ETO and GEM+VIN+CIS) were expressed as an effective combinations. Other combinations were completely ineffective. Certainly, the results require further pharmacological testing, and clinical trials.

## Strategy

How should we develop modeling approaches to identify targets in a network context? With the large amount of data generated by the new technologies, it is possible to construct the multi-dimensional molecular network in specific disease, but there are a lot of unsolved hot problems, Such as detection of disease–gene relation, characterization of disease mechanisms and process, early diagnosis of disease and individualized medical, the key points of these problem including the construction of biological networks, annotation of molecular mechanism and biological significance analysis, identification of biomarker and quantification of network intervention. Emerging evidences suggest that human diseases are associated with complex set of inner and inter factors, the inner factor refer to gene expression and gene-related regulation which could be underlined by shared TFs, histone modifications, miRNAs, and other regulators (Guan et al., 2014). The inter factors means the environmental pollution, radiation and other factor, both inner and inter factors can perturbed the normal molecular networks in human, here we name them as disease-perturbed networks, Capturing and quantifying these disease-perturbed networks is the first step in network intervention. The information gain (Info Gain) can be used to measure the total interference degree of the perturbed network. Specifically, the A gene is a variable in the perturbed network, and the entropy of A can be calculated. The B gene which regulated A gene is also a variable. If we know the information entropy of the B gene as H (B) when the A gene is disturbed. Here we need to know the joint probability distribution (Jiang et al., 2015; Zeng et al., 2016) through the data estimation and joint distribution probability formula. We set H (B| A) as the conditional entropy. The subtraction of H (B) and H (B| A) is the information gain. If the information entropy of the B gene is interfered as 1, the conditional entropy is 0.01 (the A gene is disturbed as a condition), then subtracting to 0.99, in the condition that the B gene is disturbed under the condition that A gene is disturbed, the information gain is 0.99. That is, when the A gene is disturbed. This information is important for the downstream regulation of the B gene. Therefore, we measure the total information gain for each gene in a network that can be used to characterize the perturbed network under the disease state.

**FIGURE 3:**
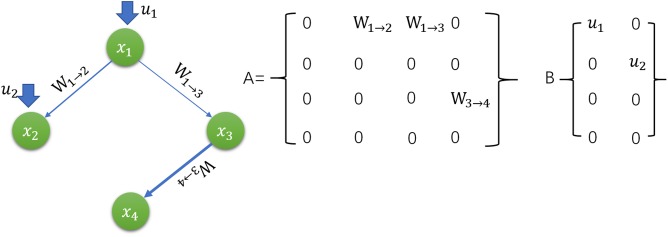
The diagram of perturbed network and intervention structure.

For a complex perturbed network, the nodes represent the genes, and the edges between the nodes represent the causal relationship of the genes. As show in **Figure 3**, we build such a system has four nodes on behalf of the four genes of the system, where *x*_1_, *x*_2_, *x*_3_, *x*_4_ represent the movement or concentration distribution, and the edges between the nodes indicates that there are interactions between the genes of the system. The weights *W*_1→2,_ *W*_1→3,_ *W*_3→4_ represent the importance of these interactions between two genes. The total edges related to one nodes means the degree of the node. The external input *u*_1_, *u*_2_ through *x*_1_ and *x*_2_ nodes to intervene the system. If the system reaches X(t) = X_f_ within a finite time or a finite concentration *t* for any given initial state x_o_ and any given final state X_f_. According to Wang and Liu (2011) and Xu et al. (2014) the system can be reversed and intervened. The system dynamics or concentration gradient equation is: *Y = A_x_ + B_u_x ∈ R^N^, u ∈ R^N^*

Where, x = x(t) = (x_1_(t),…x_N_(t) is the state of the system at time or concentration *t*, A = (a_ij_)_N×N_ is the system matrix represent the interaction in the system, for example if the distance between the individual *i* and the individual *j* is connected, then the value of the a_ij_ is the weight of the edge, otherwise a_ij_ = 0; B = (b_ij_)_N×M_,M ≤ N is the input matrix, indicating that how the external input signal (drug) controls the nodes acting in the network. And then use the Kalman rank to determine whether the network system can be intervened (Treebushny and Madsen, 2003; Liard and Lissy, 2017). For constrained systems, C = [B, AB, A^2^B,...,A^N-1^B], rank(C) = N. If the Kalman matrix C is full, the system can be intervened.

The above criteria provide a theoretical approach for the intervening performance of our criterion system, but it is a difficult task to calculate the rank of the Kalman matrix C for large-scale complex disease networks. First, for each element a_ij_ in the system matrix A, the edge weight value in the network is difficult to measure accurately; second, even if we know all the edge weights, the computational complexity is very high and difficult to apply. Thus, the study of complex interventions for complex disease network can be simplified as structural intervention study according to structure controllability by using lasso or ridge regression.

This example is designed to give a way to find the minimum number of external input required for a network intervention based on the graph theory and get the analytical value of the least input. It is found that the number of nodes that need to be intervened mainly determined by the degree distribution of the network, and the problem of determining the minimum input intervention of a directed network can be transformed into the maximum matching problem. Further study we need consider all these factors to get the best intervention for a specific disease network.

## Concluding Remarks

It is recognized that network representation of the complexity of biological systems is just the beginning, given that it just provides an overview of the system under investigation. The wisdom of target-sets, the proper concentration in the context of network properties might predominate and the patients could benefit from combinations that simultaneously impact new therapeutic strategies. Creating network intervention that addresses blindness and unthinking action in this way could, therefore, provide more benefits than multi-target therapy. As we have discussed, the application of these principles to specific diseases is in its infancy, but the early concepts are internally consistent and early results are encouraging. Attempts to confirm this hypothesis may lead to new therapeutic strategy.

## Author Contributions

A-PL, Y-HW, and CZ designed and wrote the manuscript. WZ, D-GG, and Y-HW made the model of manuscript. CZ, D-GG, and A-PL analyzed the manuscript.

## Conflict of Interest Statement

The authors declare that the research was conducted in the absence of any commercial or financial relationships that could be construed as a potential conflict of interest.

## Supplementary Material

FIGURE S1 Compare enoxaparin sodium with Isorhamnetin, Nardostachin and Jatamansin are for venous thromboembolism.

## Abbreviations

APC: *Adenomatous polyposis coli*
ASP: asparaginase
CDF: Compound Danshen Formula
CIS: Cisplatin
DOX: Doxorubicin
ETO: Etoposide
GEM: Gemcitabine
T_2_DM: type 2 diabetes mellitus
TCF: T cell factor; VIN, Vinorelbine
