# Supplementary figures and images for: Network Intervention, a Method to Address Complex Therapeutic Strategies
Source: Front Pharmacol. 2018 Jul 12;9:754. doi: 10.3389/fphar.2018.00754 (PMC6052041; doi:10.3389/fphar.2018.00754)

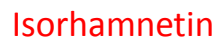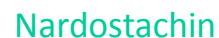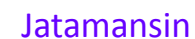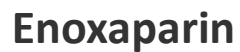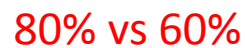

Supplement: FIGURE S1 — Compare enoxaparin sodium with Isorhamnetin, Nardostachin and Jatamansin are for venous thromboembolism. [file Image_1.PDF]
